# Mild cognitive impairment and progression to dementia in people with diabetes, prediabetes and metabolic syndrome: a systematic review and meta-analysis

**DOI:** 10.1007/s00127-018-1581-3

**Published:** 2018-09-04

**Authors:** Kingshuk Pal, Naaheed Mukadam, Irene Petersen, Claudia Cooper

**Affiliations:** 1 Research Department of Primary Care and Population Health, UCL, London, UK; 2 Division of Psychiatry, Faculty of Brain Sciences, UCL, London, UK

**Keywords:** MCI, Dementia, T2D, Systematic review, Meta-analysis

## Abstract

**Purpose:**

We aimed to quantify the relative risk of progression from mild cognitive impairment (MCI) to dementia in people with and without diabetes, and with and without the MetS (MetS); and to identify potential modifiers of the risk of progression from MCI to dementia in people with diabetes or MetS.

**Methods:**

We searched Medline, Embase, PsycINFO, PsycArticles and Web of Science from inception through to 20th March 2018. Where possible, the results from three or more studies were pooled in a meta-analysis, while other findings have been described narratively.

**Results:**

We included 15 articles reporting 12 studies (6865 participants). The overall unadjusted pooled odds ratio for the progression of MCI to dementia in people with diabetes/MetS was 1.67 (95% CI 1.27–2.19); the pooled odds ratio for progression in diabetes + MCI was 1.53 (95% CI 1.20–1.97) and in people with MetS + MCI was 2.95 (95% CI 1.23–7.05). There was moderate heterogeneity in the included studies (*I*^2^ < 60%). In diabetes, a longer duration of diabetes and the presence of retinopathy were associated with an increased risk of progression, while the use of statins and oral hypoglycaemic agents reduced the risk. Having multiple cardiovascular risk factors was a significant risk factor for progression from MCI to dementia in people with MetS.

**Conclusions:**

Diabetes and MetS were both associated with an increased incidence of dementia when co-existing with MCI. Intensive cardiovascular risk reduction and lifestyle changes for patients presenting with MCI and diabetes, prediabetes or MetS may be important in reducing incidence of dementia in this high risk population.

**Electronic supplementary material:**

The online version of this article (10.1007/s00127-018-1581-3) contains supplementary material, which is available to authorized users.

## Introduction

Demographic and lifestyle changes have seen dementia and diabetes become growing challenges to healthcare systems across the world. Dementia affects 50 million people worldwide, with the most common types of dementia being Alzheimer’s disease (AD) and vascular dementia (VaD) [1]. Less severe forms of cognitive dysfunction that precede the development of dementia affect many more people, with mild cognitive impairment (MCI) affecting 6% of the population [2], and 1 in 5 people aged 65 or older [3]. MCI is a condition that lies between age-appropriate cognition and dementia. It is defined as objective cognitive impairment relative to the person’s age, with concern about the cognitive symptoms, in a person with essentially normal functional activities who does not have dementia [4]. MCI is a heterogeneous condition with a particular subtype, amnestic MCI, linked to the development of Alzheimer’s disease [5, 6]. People with MCI are high risk for developing dementia with around 46% developing dementia within 3 years, compared to 3% of an age-matched population [7].

Diabetes has been identified as a key risk factor for dementia and MCI [4, 8, 9], so the growing prevalence of glycaemic disorders [10] has the potential to further increase the burden of MCI and dementia on healthcare systems. Understanding the links between cognitive impairment and diabetes, and the risk factors that might predict progression to dementia in people with diabetes is important in trying to mitigate such risks [11].

### MCI and risks of progression to dementia

The development of dementia is complex multifactorial degenerative process that evolves over time. People with MCI represent an important high risk group for developing dementia, especially in the context of attempts at disease modification and trying to influence the trajectory of this process [12]. The factors that play a part in this progression are a combination of discrete (non-modifiable) elements that reduce cognitive reserve (e.g. cerebrovascular events and lower educational status) and exposures that accelerate neurodegenerative processes and affect the trajectory of cognitive decline (e.g. microvascular disease and Alzheimer’s disease) [8]. This review focuses on the subset of potentially modifiable risks in people with MCI and diabetes/metabolic syndrome to identify potential targets to reduce conversion from MCI to dementia in this higher risk population.

### Disturbances of glycaemic control and metabolic syndrome as risk factors for disorders of cognition

Type 2 diabetes (T2D) has been associated with a modest increased risk in cognitive dysfunction across all cognitive domains [13]. This effect appears to be consistent across all age groups and mimics an accelerated ageing of brain function [14]. However there is also an increased risk of more severe impairment of cognition and developing dementia in older age groups that would appear to be a different phenomenon. The onset of dementia in people with T2D is on average 2.5 years earlier than in comparable populations without diabetes [11]. The relative risks of developing any cause dementia and VaD in people with T2D have been estimated to be 1.51 (95% CI 1.31–1.74) and 2.48 (95% CI 2.08–2.96), respectively [15]. Vascular damage and dysfunctions in glucose, insulin and amyloid metabolism in T2D have been proposed as mechanisms underlying this increased risk [16]. It is likely that T2D reduces cognitive reserve and increases brain susceptibility to significant insults from cerebrovascular events or dysfunctional amyloid processing.

A metabolic state that lies between normal glucose homeostasis and T2D has been defined as prediabetes [17]. Risk factors associated with prediabetes have been associated with increased dementia risk in prospective and epidemiological studies [18–21]. The prevalence of prediabetes in adult populations is rapidly rising, estimated as 35% in the UK and USA and up to 50% in China [22]. Associated with this is the metabolic syndrome (MetS)—a collection of cardiovascular risk factors that has been associated with an increased risk of developing cardiovascular disease, diabetes, mortality, and other important adverse health outcomes [23]. There are a number of different definitions for the MetS based on five cardiovascular risk factors that include abdominal obesity, hypertriglyceridemia, low high-density lipoprotein (HDL) levels, hypertension, and hyperglycaemia [24]. In research studies, a commonly used consensus definition is the presence of at least three of those risk factors [25]. Diabetes, prediabetes and MetS overlap significantly [25].

### Understanding the link between metabolic disturbances and progression of MCI to dementia

A systematic review of modifiable risk factors for the progression of MCI to dementia identified diabetes and prediabetes as important predictors [4]. We assimilate below current evidence that may explain this relationship, or identify modifiers of this increased dementia risk in people with T2D, prediabetes and MetS.

#### Cardiovascular and metabolic risks

*Hypertension* Raised blood pressure has been identified as a risk factor for developing dementia in older people with T2D in cohort studies [26, 27]. However, findings from a systematic review did not find that hypertension predicted progression from any-type MCI to dementia in the general population [4]. A Dutch cohort study found an association between slight cognitive decline and higher blood pressure in the prediabetes stage [28].

*Adiposity* Midlife total body adiposity and central adiposity have been associated with increased risks of dementia and are also common in people with T2D, prediabetes and MetS. Results from a prospective cohort study of 10,276 people in the USA found a hazard ratio for developing dementia of 1.74 in people with a body mass index (BMI) ≥ 30 and a hazard ratio of 1.35 (95% CI 1.14–1.60) in people with a BMI of 25–29.9 [21]. A systematic review found an association between high BMI and increased risk of dementia in five out of nine studies [19].

*Cholesterol* Increased blood cholesterol levels in midlife (but not later life) are associated with increased dementia risk in the general population [19, 29]. In people living with T2D, results have been mixed with dyslipidaemia being associated with both increased and decreased risks of cognitive impairment [27, 30]. Cholesterol levels in later life do not appear to increase the risk of progression from any-type MCI to all-cause dementia [4].

*Glycaemic control* There are four main aspects of glycaemic control that have been reported to be associated with dementia risk in people living with diabetes—duration of diabetes, blood glucose control (e.g. HbA1c and fasting plasma glucose), use of medication and episodes of hypoglycaemia. An increase in dementia risk of 40–60% in people who have been living with diabetes for 5 years or more relative to those more recently diagnosed is reported [30, 31]. Higher mean blood glucose readings may be associated with an increased risk of dementia [32], but not in older patients over 85 [33]. The use of oral hypoglycaemic agents (and statins), but not insulin, has been linked to a lower risk of developing dementia [31]. Hypoglycaemia appears to have a bi-directional association with cognitive impairment [34–36].

### Other modifiable risk factors for cognitive decline that have been highlighted in previous reviews

There is some evidence from studies looking at risk factors for cognitive decline that diet, physical activity, smoking and depression may affect the rate of cognitive decline. A Mediterranean diet has been associated with lower risks of developing cognitive disorders and reduced rate of progression to dementia in recent meta-analyses [37, 38]. There is low quality evidence from observational studies in the general population that the risk of dementia is lowered by omega-3 fatty acids and vegetable intake [39]. In a recent review, an increase in leisure-time physical activity was associated with a 10% reduction in dementia risk [40]. In people with T2D, there is evidence that suggests physical activity may not affect the risk of cognitive decline [41], but a low intake of saturated and trans-fat, and a high intake of polyunsaturated fat since midlife has been associated with reduced cognitive decline [42]. In the general population, heavy smoking in mid-life more than doubles the risk of developing dementia and people actively smoking in later life have a higher risk of incident dementia [43, 44].

Depression is another potentially important factor, with depression affecting up to 39% of people living with T2D [45] and people with T2D and depression being twice as likely to develop dementia [46, 47].

This review will update and synthesise the most recent evidence from longitudinal observation studies describing modifiable risk factors that predict the progression of MCI to dementia in people living with T2D, prediabetes or MetS.

## Methods

We used searched for the relevant literature in Medine, Embase, PsycINFO, PsycArticles and Web of Science from inception through to 20.3.18. No limits were set for language or date of publication. References of included articles and relevant reviewed were also searched. The search strategy can be found in ESM Appendix 1.

We included longitudinal studies involving people living with T2D, prediabetes or MetS diagnosed with MCI. MCI was defined as cognitive impairment identified from objective neuropsychological tests, in the absence of dementia or significant functional impairment. Studies recruited from either the general population, or from clinical settings where MCI had already been diagnosed. Modifiable risk factors were risks that could be influenced by changes in lifestyle or medical treatment.

The exclusion criteria for studies were: (1) cross-sectional studies, (2) studies not reporting the outcome measures of interest, (3) proceedings from conferences not published in peer-reviewed journals, and (4) studies on patients with diabetes where the mean age of participants was < 60 if type of diabetes not specified.

### Data extraction and quality assessment

Two authors (KP, NM) independently extracted study characteristics and findings into specific data extraction tables. Any disagreements were resolved by discussion with a third author (CC). The risk of bias was independently evaluated by the same authors by comparison against criteria based on previously published checklists [4, 16]. Studies were given a quality score out of 10 based on criteria listed in ESM Appendix 2 with higher scores indicating higher quality. Studies were rated 0–2 across five domains: population selection and recruitment, participation at follow-up, diabetes and MetS assessment, dementia assessment and data analysis.

### Analysis

Analysis started with a narrative synthesis of the data. Heterogeneity of methods, outcomes and populations were assessed to determine the appropriateness of subsequent meta-analysis. Where the data allowed, the results from three or more studies were pooled using a random-effects model and pooled odds ratios for binary outcomes (progression to dementia/no progression). The meta-analysis was done using RevMan 5.3 from the Cochrane Collaboration. Where meta-analysis was not possible, the findings have been described narratively.

## Results

### Overview of included studies

**Fig. 1:**
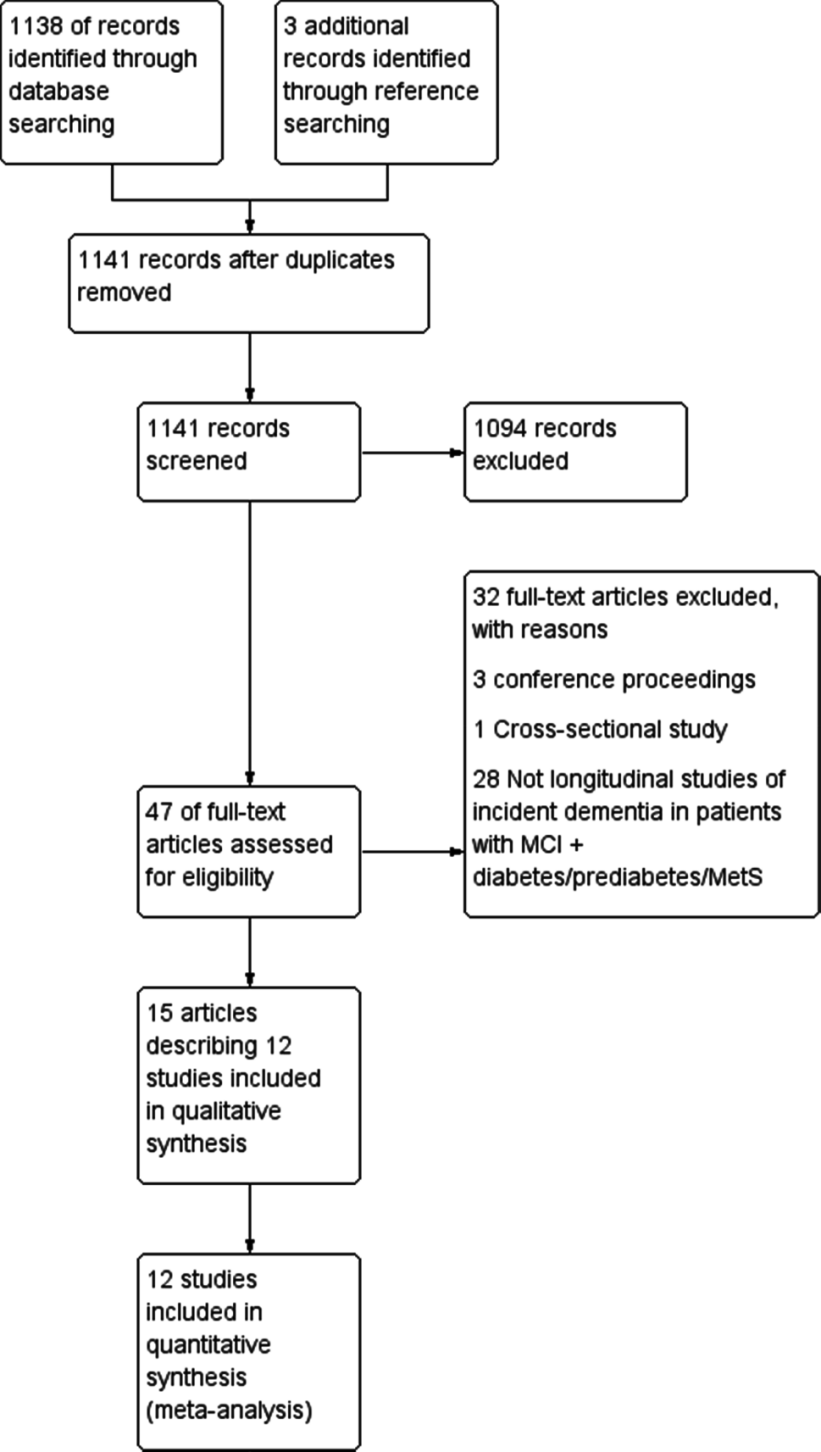
PRISMA flow diagram of study

The results of our search strategy have been summarised in a PRISMA flow diagram in Fig. 1. Details of the included studies can be found in Table 1 and this has been summarised below. We included 15 articles reporting 12 studies with 6865 participants [48–59]. Eight studies only included people with diabetes or MetS, while data for patients with diabetes were extracted from reports of four studies. Half of the studies were community-based and half were clinical studies. Eight studies included people with diabetes, three studies featured participants with MetS and one study reported outcomes for both diabetes and MetS. Three studies took place in Italy, two in China, two in Singapore and the remainder in France, the Netherlands, Sweden, the UK and the USA. Three studies looked at the risk of progression of MCI to AD, while nine studies looked at progression to all cause dementia. There was moderate heterogeneity in the studies included in the main meta-analysis with an overall *I*^2^ statistic of 52%. A funnel plot of the studies has been included in ESM Appendix 3. The plot appears mostly symmetrical; however, there is some asymmetry near the base suggesting the absence of lower powered studies with negative results which may raise the possibility of publication bias.

**Table 1:** Summary of included studies

Study	Country	Recruitment	Clinic/community	Diabetes/metabolic syndrome	Duration of FUP	Outcomes	Quality score (/10)
Artero [49]	France	Random sample recruited from French electoral roles	Community	Diabetes	4 years	All cause dementia	10
Ciudin [50]	Italy	Patients attending a memory clinic, Fundacio ACE, aged > 60 with type 2 diabetes	Clinic	Diabetes	2 years	All cause dementia	4
Exalto et al. [51]	Netherlands	Recruited from a memory clinic based Amsterdam Dementia Cohort of VU University Medical Centre	Community	Metabolic syndrome^a^	0.6–4.6 years	All cause dementia	8
Li et al. [52]	China	Subjects were sampled from ten randomly selected communities in the city of Chongqing	Clinic	Diabetes	5 years	Conversion to Alzheimer’s dementia	8
Ma et al. [53]	China	Recruitment from six geographically convenient communities with high proportions of elderly residents within Tianjin city, China	Community	Diabetes	4 years	All cause dementia	7
Morris et al. [54]	USA	Data were obtained from ADNI on 5 January 2012. ADNI is conducted by the National Institute on Aging, the National Institute of Biomedical Imaging and Bioengineering, pharmaceutical companies, and nonprofits	Clinic	Diabetes	2 years	Conversion to Alzheimer’s dementia	8
Ng et al. [55]	Singapore	Participants were of Chinese ethnicity and recruited from five districts in the South East region of Singapore from September 1, 2003 to December 31, 2009	Community	Metabolic syndrome^b^ and diabetes	4 years	All cause dementia	8
Prasad et al. [56]	Singapore	Retrospective analyses of a prospective clinical database comprising patients with cognitive impairment managed at the memory clinic of a tertiary neurology center between January 2008 and January 2011	Clinic	Diabetes	Minimum 18 months	Conversion to Alzheimer’s dementia	4
Ravaglia et al. [57]	Italy	Participants were recruited among the outpatients seeking medical advice for cognitive complaints at the Center for Physiopathology of Aging, University of Bologna	Clinic	Diabetes	From 6 months to 5 years	All cause dementia	4
Solfrizzi et al. [58]	Italy	A sample of 5632 subjects aged 65–84 years, independent or institutionalized, was randomly selected from the electoral rolls of eight Italian municipalities, after stratification for age and gender	Community	Metabolic syndrome^a^	3 years	All cause dementia	7
Velayudhan et al. [59]	UK	Potential candidates were identified from general practice registers and invited to participate. Participants were assessed annually from 2001 to 2007	Clinic	Diabetes	4 years	All cause dementia	5
Xu et al. [48]	Sweden	Participants recruited from all registered inhabitants who were age 75 years or older and living in the Kungsholmen district of central Stockholm, Sweden, in 1987	Community	Diabetes	9 years	All cause dementia	6

^a^MetS diagnosis: ATPIII criteria—3 or more of the following components abdominal obesity (waist circumference > 102 cm for men and > 88 cm for women); elevated plasma triglycerides (≥ 150 mg/dL); low HDL cholesterol (< 40 mg/dL for men and < 50 mg/dL for women); high blood pressure(≥ 130/≥85 mmHg) or being in hypertensive treatment; high fasting plasma glucose (≥ 110 mg/dL)

^b^MetS diagnosis: International Diabetes Federation criteria—central obesity (waist circumference ≥ 90 cm for men and ≥ 80 cm for women) plus at least 2 CVRFs, including raised triglyceride levels (≥ 150 mg/dL) or specific treatment for this lipid abnormality; reduced high-density lipoprotein cholesterol level (< 40 mg/dL in men and < 50 mg/dL in women) or specific treatment for this lipid abnormality; raised blood pressure (systolic ≥ 130 mm Hg or diastolic ≥ 85 mm Hg or treatment of previously diagnosed hypertension) and raised fasting plasma glucose level (≥ 100 mg/dL or previously diagnosed type 2 diabetes mellitus)

### Quality of included studies

Most of the studies were of moderate to high quality based on the criteria described above (scoring 5 or higher). The study quality scores are summarised in Table 1. No single criterion was judged to be essential and no studies were excluded based on scores. Lower scoring study reports contained little detail regarding the population sample or response rates for inclusion in the study, and the diagnosis of diabetes was based on medical records rather than direct measurement.

#### Impact of metabolic status on risk of progression of MCI to dementia

**Fig. 2:**
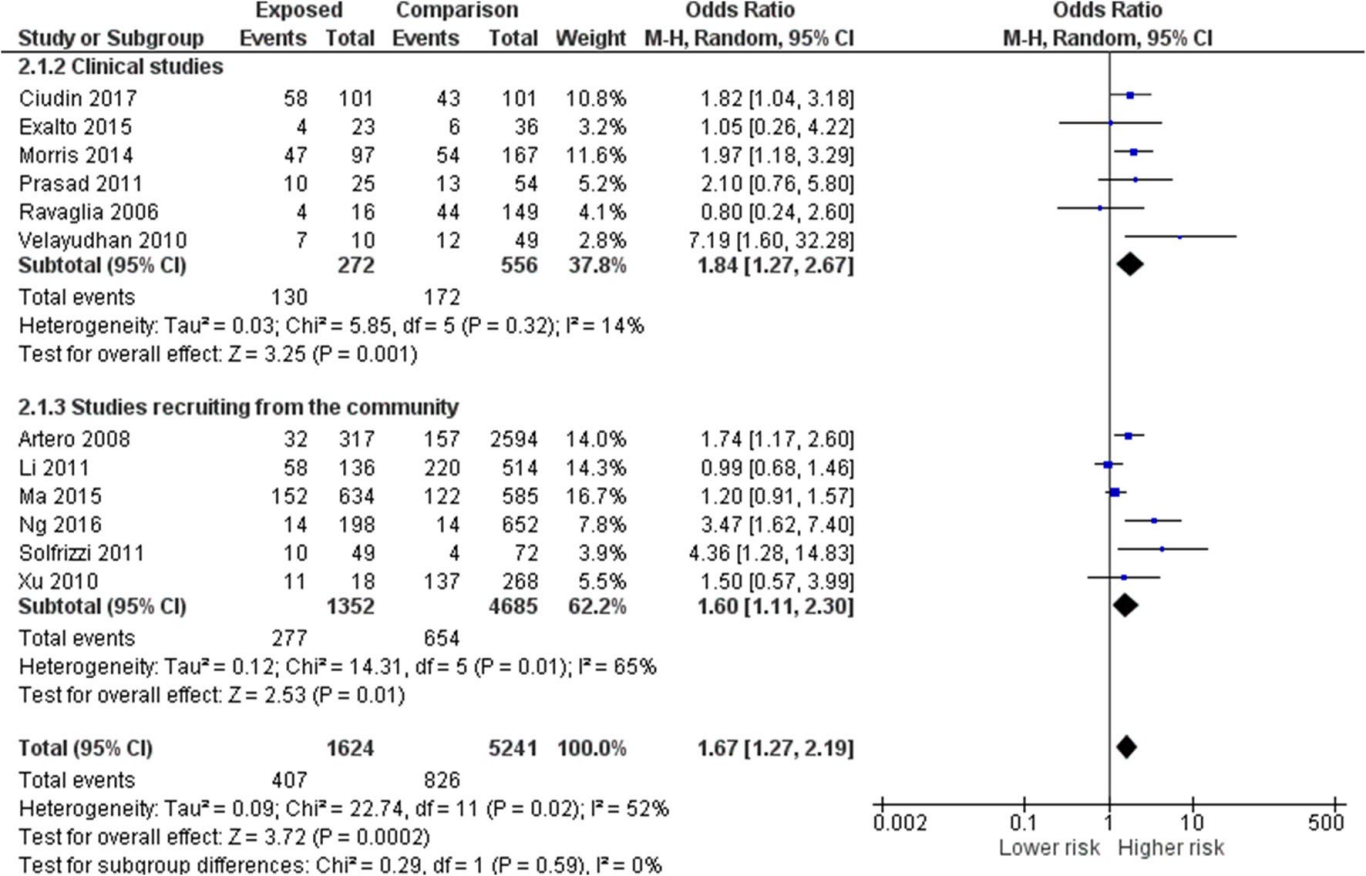
Meta-analysis of pooled odds ratios of risk of progression from MCI to dementia in people with diabetes, prediabetes or metabolic syndrome

**Fig. 3:**
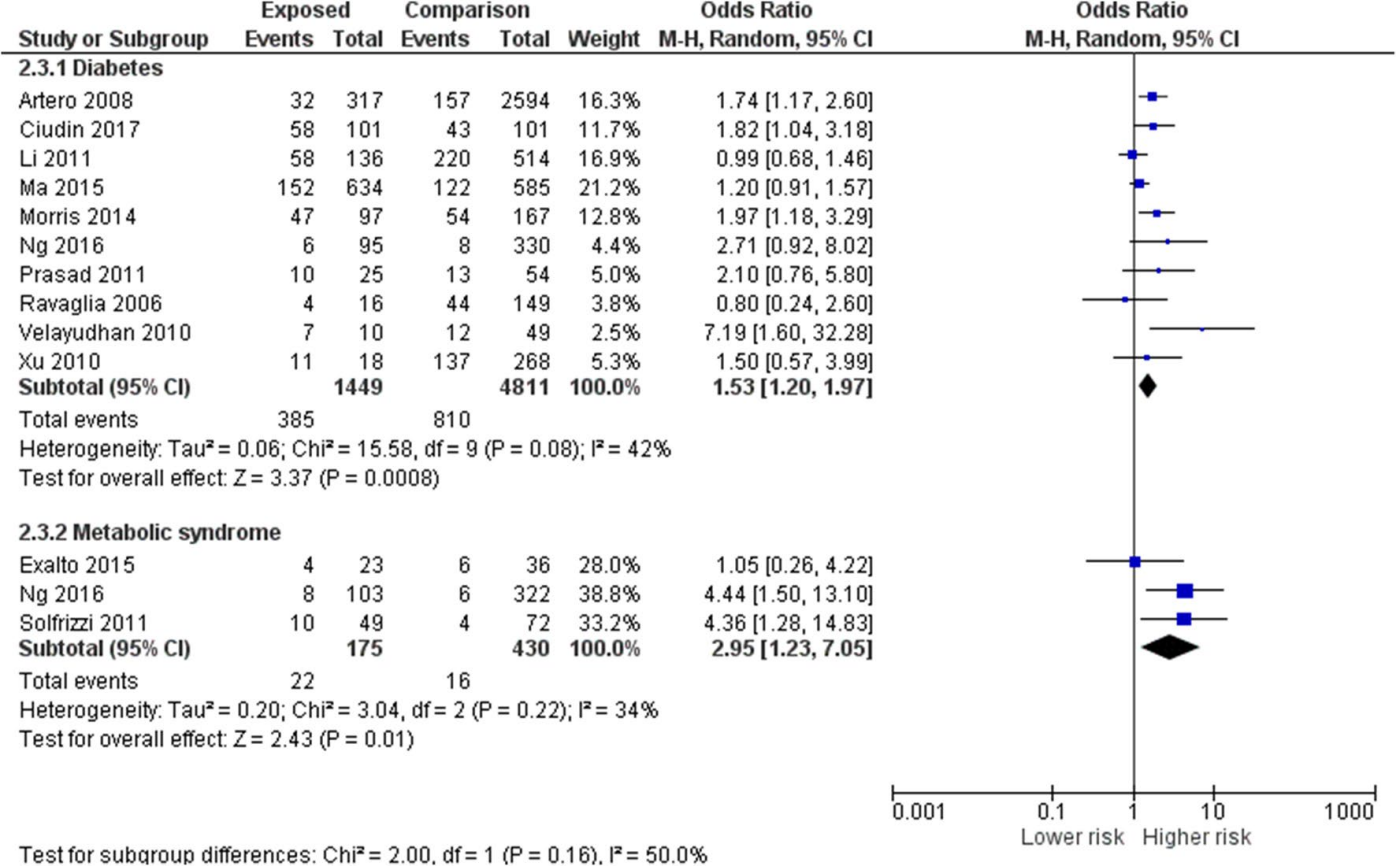
Subgroup analysis comparing pooled odds ratios of risk of progression from MCI to dementia in people with diabetes and metabolic syndrome

Figure 2 shows the overall the unadjusted pooled odds ratio for the progression of MCI to dementia in people with diabetes or MetS from 12 studies was 1.67 (95% CI 1.27–2.19). The risk was similar in studies that recruited from memory clinics (pooled OR from six studies 1.84, 95% CI 1.27–2.67) compared to epidemiological studies [pooled OR from six studies 1.60, (95% CI 1.11–2.30)]. Figure 3 shows that the pooled odds ratio for progression in people with diabetes was 1.53 (95% CI 1.20–1.97) while the pooled odds ratio in people with MetS was 2.95 (95% CI 1.23–7.05). Two studies that separated the risks of diabetes and prediabetes/MetS also found a trend towards a higher risk for people with prediabetes and MetS [48, 55]. The adjusted HR for all cause dementia in one of the studies was 4.96 (95% CI 2.27–10.84) in people with prediabetes, nearly double the HR of 2.87 (95% CI 1.30–6.34) in people with T2D; similarly the adjusted HR for MetS in the other study was 4.25 (95% CI 1.29–14.00), while the adjusted HR for people with diabetes was 2.47 (95% CI 1.92–4.19).

#### Type of dementia

Four studies provided a breakdown of the type of dementia diagnosed when participants progressed from MCI [48, 50, 53, 59]. Data from all four studies were from people with diabetes. In three out of the four studies, the most common diagnosis was AD with the proportion of people diagnosed with AD varying between 23 and 84%. One study reported VaD to be the most common diagnosis (46%) while the range of diagnosis of VaD in other studies was between 4 and 14%. Mixed dementia was diagnosed in 4–11% of participants and Lewy body dementia was diagnosed in 1–7% of participants.

#### Time to diagnosis

Three studies reported times to diagnosis of dementia in people living with diabetes who had MCI [48, 50, 53]. The time to diagnosis was shorter for people with diabetes compared to people without in all three studies, with a median time to diagnosis of 1.83–1.97 years, accelerated by 4 months–3 years.

### Risk factors for progression of MCI to dementia in people with diabetes and MetS

#### Cardiovascular and metabolic risks

*Hypertension* In one study, the risk of incident dementia appeared to be nearly doubled in the presence of hypertension but this did not reach statistical significance (adjusted HR 1.84 with 95% CI 0.55–6.22). In the other study, the rate of incident dementia per 1000 person-years was four times higher in people with MetS who had hypertension when compared to people without: 72.21 (95% CI 36.11–144.39) for hypertension and MetS compared to 17.49 (95% CI 4.37–69.92) for people with hypertension without MetS.

*Central obesity* Similar to the results above, the adjusted HR for central obesity was nearly three times higher, without reaching statistical significance (adjusted HR 2.97 with a 95% CI of 0.85–10.40) with an incident dementia rate of 80.93 per 1000 person-years (95% CI 42.11–155.54).

*Dyslipidaemia* Like the previous two results, the adjusted HR for dyslipidaemia also did not reach statistical significance (2.04 with a 95% CI 0.61–6.78)—however, there seemed to be a marked difference between incident dementia rates associated with high triglycerides compared with low HDL. The rate of incident dementia per 1000 patient-years in MetS was calculated to be 84.53 (95% CI 37.98–188.16) with high triglycerides compared to 42.96 (16.12–114.46) for low HDL cholesterol.

*Multiple cardiovascular risk factors* Both the above studies looked at people with MetS and therefore looked at the impact of having three or more risk factors out of hypertension, central obesity, dyslipidaemia and hyperglycaemia. People with three or more risk factors were nearly five times more likely to progress from MCI to dementia (adjusted HR 4.92 95% CI 1.39–17.40). The incident rate of progression to dementia with three or more risks factors was 67.6 (95% CI 35.17–129.93).

*Statin use* One of the other included studies explored the impact of statins on the risk of progressing from MCI to dementia in people with diabetes [53]. The use of a statin was associated with a lower risk of progression with an adjusted HR of 0.86 (95% CI 0.84–0.90).

*Glycaemic control* Two studies on people with diabetes looked at the impact of aspects of glycaemic control on the risks of MCI progressing to dementia [50, 53]. A longer duration of diabetes was associated with an increasing risk of developing dementia. The adjusted HRs increased from 1.04 (95% CI 09.98–1.10) after 2 years of living with diabetes to 1.42 (95% CI 1.35–1.49) after more than 5 years. An HbA1c of 7% or more was associated with an increased risk of dementia with a HR of 1.30 (95% CI 1.11–1.57). Using insulin did not affect the risk of dementia, but oral hypoglycaemic agents appeared to reduce the risk of developing dementia (HR 0.93, 95% CI 0.90–0.96). Patients with diabetes who converted from MCI to dementia were more likely to have diabetic retinopathy and had reported more episodes of severe hypoglycaemia.

In patients with MetS, high blood glucose levels were associated with an incident rate of dementia of 14.63 (95% CI 2.06–103.88) per 1000 person-years—the lowest calculated incident rate of the five features or MetS [58].

### Other risk factors for the progression of MCI to dementia in people with diabetes, prediabetes and MetS

None of the studies in this review reported data on the impact of diet, physical activity or depression in the progression of MCI to dementia in people with T2D, prediabetes or MetS. There was some evidence on non-modifiable risks which we will only briefly summarise as this was not the focus of this review. One study reported age as a major risk factor with the risk of progression to dementia increasing dramatically with age and people with diabetes and MCI aged 75–85 had twice the dementia risk of people aged 65–75 [53]. There was no evidence of significant risks from other lifestyle or demographic factors. Two studies reported no significant increase in the risk of converting to dementia in people with diabetes from gender, education level, smoking, heavy drinking or previous cerebrovascular disease [50, 53]. The APOEε4 allele was found to be associated with an increase in the risk of progressing to dementia in two studies [50, 54], but not in one study in a younger Chinese population [53].

## Discussion

We have synthesised evidence on diabetes, prediabetes and MetS and other cardiovascular risk factors with regards to risk of progressing from MCI to dementia. We used a thorough and inclusive search strategy with no limitations on language or date of publication. Our results are likely to represent the most up to date and comprehensive overview of this topic.

### Summary of results

Diabetes, prediabetes and MetS were all associated with increased risks of progression of MCI to dementia. The pooled odds ratio for progression in people with diabetes was 1.53 (95% CI 1.20–1.97) while the pooled odds ratio in people with MetS was 2.95 (95% CI 1.23–7.05). In people with T2D, a longer duration of diabetes and the presence of retinopathy were associated with an increased risk of progression from MCI to dementia, while statins and oral hypoglycaemic agents appeared to reduce the risk. For people with MetS, the presence of multiple cardiovascular risk factors was a significant risk factor for progression from MCI to dementia. The highest rates of incident dementia were associated with raised triglycerides, abdominal obesity and hypertension, with lower rates associated with low HDL cholesterol and raised blood glucose levels. Overall, most of the studies included in this review tended towards a higher risk of progression to dementia in people with diabetes or MetS. Two studies reported results that tended towards a lower risk of progression of MCI to dementia in people with diabetes, and one of those was rated high quality [52, 57]. The higher quality study looked at rates of Alzheimer’s disease in a Chinese population [52]. Based on the results from other studies in a similar population, this study may have only detected half of all cause dementia (particularly missing cases of VaD), and therefore the risk of progression to dementia may have appeared significantly lower than other studies.

### Comparison to previous literature

There was conflicting evidence in this review on the role of APOEe4 in the progression of MCI to dementia with one study reporting no evidence of links between APOEe4 and progression to dementia [53]. This study involved a community Chinese population and it was also the only study that showed a higher risk of progression to VaD than Alzheimer’s disease. Therefore, the pathways for progression of MCI to dementia might be different in different ethnic groups and the type of dementia developed may vary.

There was no clear evidence for the impact of individual cardiovascular risk factors on the rate of progression of MCI to dementia. This is similar to findings in the general population where individual risk factors such as hypertension or hypercholesterolaemia have not been shown to increase the rate of progression of MCI to dementia [4]. However, when three or more risk factors clustered together as MetS, the risks increased substantially. This may represent a cumulative effect or be due to other pathology associated with MetS that might include chronic inflammation, insulin-resistance and the endocrine influence of adipose tissue [23].

The pooled OR for progression of MCI to dementia in people with MetS tended towards being higher than people with diabetes. This might be due to more active treatment of risk factors such as hypertension and raised cholesterol in patients with diabetes. Renin–angiotensin system drugs are very commonly used in diabetes for treating hypertension and micro-albuminuria, and these medicines have been shown to be protective against cognitive impairment [30].

With regards to dyslipidaemias, previous studies have focused on hypercholesterolaemia with no evidence that raised cholesterol in later life affects dementia risk [4, 60]. However, statins have previously been shown to substantially lower the risk of dementia [61]—this effect was endorsed by another study included in this review [53] and patients with T2D are likely to be on statins because of their raised cardiovascular risk and lower target cholesterol levels. The particular dyslipidaemia associated with MetS may also point to a more significant role for raised triglycerides in the aetiology of dementia in this group.

Oral hypoglycaemic drugs (but not insulin) were also found to have beneficial effects in this review, so the combination of multiple treatments for multiple risk factors in people with T2D may explain why the risks in this group are comparatively lower than prediabetes or MetS. Pursuing a similar pro-active treatment of risk factors in prediabetes or MetS could help with a 10–25% reduction in risk factors that could prevent more than a million cases of dementia worldwide [29].

### Implications for practice

The results of this review have identified a number of potential risk factors that could be targeted to reduce or slow down the progression of MCI to dementia in people with the MetS or diabetes. Optimising the treatment of cardiovascular risk factors in people with MetS may be a potentially important therapeutic opportunity. Promoting the use of statins and oral hypoglycaemic agents in patients with T2D and MCI may also be important, and needs to be weighed against the recent trend towards relaxing HbA1c targets in older people with T2D. Tight control of blood glucose levels with an HbA1c ≤ 48 mmol/mol (7%) could potentially have a meaningful impact by delaying progress to dementia that could happen within 2 years of a diagnosis of MCI.

### Limitations of research

There may have been risk factors that were analysed in studies but not reported so we may have under-reported null results not described in the published articles. Most of the risk factors for progression were described in studies on patients with MetS and there was a lack of studies looking at risk factors for progression in patients with T2D. From the study reports it was also difficult to distinguish between treated and untreated risk factors. Diagnosing dementia and the types of dementia can be challenging and may have affected the accuracy of the findings. The process of measuring the conversion of MCI to dementia has limitations as the only difference between MCI and mild dementia may be the interpretation of the impact of the condition on activities of daily living and may be at risk of bias [62]. Additionally, diabetes itself could impact on a person’s function over time and contribute to frailty which makes attribution of MCI progression to risk factors even more difficult. Only 4 out of 12 studies provided details of the type of dementia diagnosed.

### Suggestions for future research

There are a number of questions regarding the development of cognitive disorders in diabetes, prediabetes and MetS raised by this review. More research is needed on the role of APOEe4 in different ethnicities and whether this impacts the type of dementia that develops from MCI. Most of the studies included in this review did not distinguish between aMCI and non-aMCI, and given the significant variation in rates of progression to Alzheimer’s disease across the studies, it would be useful to know if this was reflected in the preceding MCI stage. The role of raised triglycerides in developing dementia is potentially under-researched and may be important in people with MetS. We did not find any studies that looked at the impact of identifying or treating depression in patients with MCI and diabetes, prediabetes or MetS.

Developing and evaluating multi-modal interventions to harness lifestyle and therapeutic strategies to target modifiable risk factors and reduce the progression of MCI to dementia in these high risk groups may have the potential for significant patient benefit. However, a key question for such interventions would be the optimal timing for delivery—whether treatment can be effective after the development or MCI, or whether interventions are needed in mid-life. It would also be helpful for studies to report more details about outcome measures regarding conversion of MCI to dementia. Quantifiable changes in cognition and more details about subsequent dementia diagnoses would help distinguish between progression of cognitive impairment and worsening frailty which would support better understanding of the nature of progression and provide more robust outcomes less reliant on subjective interpretation.

## Conclusion

Diabetes, prediabetes and MetS were all associated with increased risks of progression of MCI to dementia. The pooled odds ratio for progression in people with diabetes was 1.53 (95% CI 1.20–1.97) while the pooled odds ratio in people with MetS was 2.95 (95% CI 1.23–7.05). In people with T2D, a longer duration of diabetes and the presence of retinopathy were associated with an increased risk of progression from MCI to dementia, while statins and oral hypoglycaemic agents appeared to reduce the risk. For people with MetS, the presence of multiple cardiovascular risk factors was a significant risk factor for progression from MCI to dementia. Intensive cardiovascular risk reduction and lifestyle changes for patients presenting with MCI and diabetes, prediabetes or MetS may be important in reducing the incidence of dementia in this high risk population.

## Electronic supplementary material

Below is the link to the electronic supplementary material.

Supplementary material 1 (DOCX 33 KB)
