# Supplementary material for: Mild cognitive impairment and progression to dementia in people with diabetes, prediabetes and metabolic syndrome: a systematic review and meta-analysis
Source: Soc Psychiatry Psychiatr Epidemiol. 2018 Sep 4;53(11):1149–60. doi: 10.1007/s00127-018-1581-3 (PMC6208946; doi:10.1007/s00127-018-1581-3)

**Appendix 1 – search terms and strategy**

**Population – prediabetes or type 2 diabetes:**

- Type 2 diabetes
- NIDDM
- T2DM
- T2D
- Prediabetes or pre-diabetes
- Impaired glucose tolerance or IGT
- Impaired fasting glycaemia or IFG
- Metabolic syndrome
- Insulin resistance

AND{

**Condition - MCI:**

- MCI
- mild cognitive
- cognitive impairment
- benign senescent forgetfulness
- age associated cognitive decline
- age-associated memory impairment
- age-related cognitive decline
- mild neurocognitive disorder

*AND*{

**Outcome:**

- Dementia incidence
- Incident dementia
- Incidence of dementia

*OR*

**Study type:**

- Prospective
- Cohort
- Longitudinal

**Search for MEDLINE**

# Searches Annotations Actions

*1 type 2 diab$.mp. [mp=ti, ab, hw, tn, ot, dm, mf, dv, kw, fx, nm, kf, px, rx, an, ui, sy, tc, id, tm]*

*2 NIDD$.mp. [mp=ti, ab, hw, tn, ot, dm, mf, dv, kw, fx, nm, kf, px, rx, an, ui, sy, tc, id, tm]*

*3 T2D$.mp. [mp=ti, ab, hw, tn, ot, dm, mf, dv, kw, fx, nm, kf, px, rx, an, ui, sy, tc, id, tm]*

*4 Prediabetes.mp. [mp=ti, ab, hw, tn, ot, dm, mf, dv, kw, fx, nm, kf, px, rx, an, ui, sy, tc, id, tm]*

*5 Prediab$.mp. [mp=ti, ab, hw, tn, ot, dm, mf, dv, kw, fx, nm, kf, px, rx, an, ui, sy, tc, id, tm]*

*6 pre-diabetes.mp. [mp=ti, ab, hw, tn, ot, dm, mf, dv, kw, fx, nm, kf, px, rx, an, ui, sy, tc, id, tm]*

*7 pre-diab$.mp. [mp=ti, ab, hw, tn, ot, dm, mf, dv, kw, fx, nm, kf, px, rx, an, ui, sy, tc, id, tm]*

*8 Impaired glucose tolerance.mp. [mp=ti, ab, hw, tn, ot, dm, mf, dv, kw, fx, nm, kf, px, rx, an, ui, sy, tc, id, tm]*

*9 IGT.mp. [mp=ti, ab, hw, tn, ot, dm, mf, dv, kw, fx, nm, kf, px, rx, an, ui, sy, tc, id, tm]*

*10 impaired fasting glycaemia.mp. [mp=ti, ab, hw, tn, ot, dm, mf, dv, kw, fx, nm, kf, px, rx, an, ui, sy, tc, id, tm]*

*11 ifg.mp. [mp=ti, ab, hw, tn, ot, dm, mf, dv, kw, fx, nm, kf, px, rx, an, ui, sy, tc, id, tm]*

*12 metabolic syndrome.mp. [mp=ti, ab, hw, tn, ot, dm, mf, dv, kw, fx, nm, kf, px, rx, an, ui, sy, tc, id, tm]*

*13 insulin resistance.mp. [mp=ti, ab, hw, tn, ot, dm, mf, dv, kw, fx, nm, kf, px, rx, an, ui, sy, tc, id, tm]*

**14 or/1-13**

*15 MCI.mp. [mp=ti, ab, hw, tn, ot, dm, mf, dv, kw, fx, nm, kf, px, rx, an, ui, sy, tc, id, tm]*

*16 mild cognitive.mp. [mp=ti, ab, hw, tn, ot, dm, mf, dv, kw, fx, nm, kf, px, rx, an, ui, sy, tc, id, tm]*

*17 cognitive impairment.mp. [mp=ti, ab, hw, tn, ot, dm, mf, dv, kw, fx, nm, kf, px, rx, an, ui, sy, tc, id, tm]*

*18 benign senescent forgetful$.mp. [mp=ti, ab, hw, tn, ot, dm, mf, dv, kw, fx, nm, kf, px, rx, an, ui, sy, tc, id, tm]*

*19 age associated cognitive decline.mp. [mp=ti, ab, hw, tn, ot, dm, mf, dv, kw, fx, nm, kf, px, rx, an, ui, sy, tc, id, tm]*

*20 age associated memory impairment.mp. [mp=ti, ab, hw, tn, ot, dm, mf, dv, kw, fx, nm, kf, px, rx, an, ui, sy, tc, id, tm]*

*21 age related cognitive decline.mp. [mp=ti, ab, hw, tn, ot, dm, mf, dv, kw, fx, nm, kf, px, rx, an, ui, sy, tc, id, tm]*

*22 mild neurocognitive disorder.mp. [mp=ti, ab, hw, tn, ot, dm, mf, dv, kw, fx, nm, kf, px, rx, an, ui, sy, tc, id, tm]*

**23 or/15-22**

*24 dementia incidence.mp. [mp=ti, ab, hw, tn, ot, dm, mf, dv, kw, fx, nm, kf, px, rx, an, ui, sy, tc, id, tm]*

*25 dementia inc$.mp. [mp=ti, ab, hw, tn, ot, dm, mf, dv, kw, fx, nm, kf, px, rx, an, ui, sy, tc, id, tm]*

*26 incident dementia.mp. [mp=ti, ab, hw, tn, ot, dm, mf, dv, kw, fx, nm, kf, px, rx, an, ui, sy, tc, id, tm]*

*27 inc$ dementia.mp. [mp=ti, ab, hw, tn, ot, dm, mf, dv, kw, fx, nm, kf, px, rx, an, ui, sy, tc, id, tm]*

**28 or/24-27**

*29 prospective.mp. [mp=ti, ab, hw, tn, ot, dm, mf, dv, kw, fx, nm, kf, px, rx, an, ui, sy, tc, id, tm]*

*30 cohort.mp. [mp=ti, ab, hw, tn, ot, dm, mf, dv, kw, fx, nm, kf, px, rx, an, ui, sy, tc, id, tm]*

*31 longitudinal.mp. [mp=ti, ab, hw, tn, ot, dm, mf, dv, kw, fx, nm, kf, px, rx, an, ui, sy, tc, id, tm]*

**32 or/29-31**

**33 14 and 23 and (28 or 32)**

**Appendix 2: Quality scoring criteria**

Population selection and recruitment (maximum 2 points)

- Well-defined population sample (1 point)
- Baseline response rate 70% or more (1 point)

Participation at follow-up (maximum 2 points)

- Participation rate at follow-up 60–69·9% (1 point), OR
- 70% response or more (2 points)

Diabetes/metabolic status assessment (maximum 2 points)

- Based on medical records/self-reporting (0 points),
- Random glucose measurement (1 point), OR
- Diabetes diagnosed by fasting glucose/oral glucose tolerance test / HbA1c (2 points)
- MetS diagnosed by measurements of blood lipids, blood glucose and waist circumference (2 points)

Dementia assessment and diagnosis (maximum 2 points)

- Based on medical records (0 points),OR
- Active screening with ad-hoc criteria (1 point), OR

On recognised international criteria by a central consensus committee (2 points)

Data analysis (maximum 2 points)

- Analysis excluded those with dementia at baseline, and included adjustment for confounders (1 point)
- Prospective analysis with estimation of standard error taking design features into account (1 point)

Appendix 3: Funnel plot for main meta-analysis


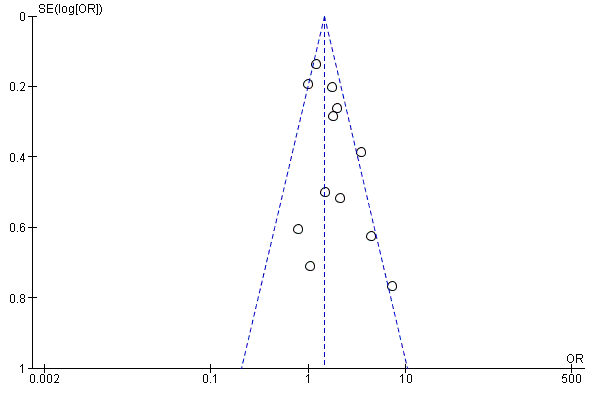

Supplement: Supplementary file 1 — Supplementary material 1 (DOCX 33 KB) [file 127_2018_1581_MOESM1_ESM.docx]
